# Supplementary material for: Reactions of Cadmium(II) Halides and Di-2-Pyridyl Ketone Oxime: One-Dimensional Coordination Polymers
Source: Molecules. 2024 Jan 19;29(2):509. doi: 10.3390/molecules29020509 (PMC10820575; doi:10.3390/molecules29020509)
Supplement: Supplementary file 1 [file molecules-29-00509-s001.zip › molecules-2784410-supplementary.pdf]

Supplementary Materials:

Reactions of Cadmium(II) Halides and Di-2-pyridyl Ketone Oxime: One-dimensional Coordination Polymers

Christina Stamou, Pierre Dechambenoit, Zoi G. Lada, Patroula Gkolfi, Vassiliki Riga, Catherine P. Raptopoulou, Vassilis Psycharis, Konstantis F. Konidaris, Christos T. Chasapis and Spyros P. Perlepes

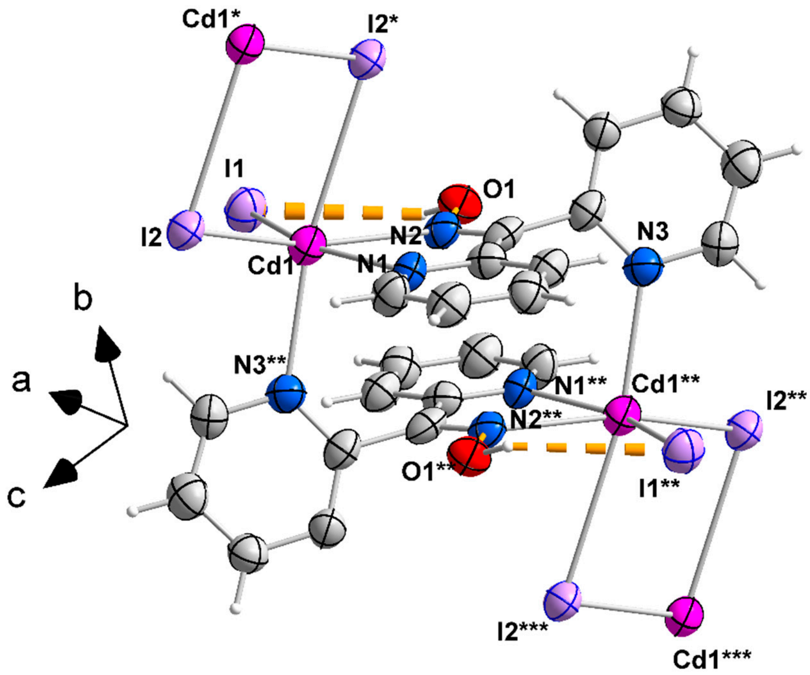

Figure S1. Complete coordination spheres of two neighboring Cd^{II} centers (Cd1, Cd1**) in one zigzag chain of complex **3**. The symmetry codes are the same with those mentioned for compound **2** in Figure 2b.

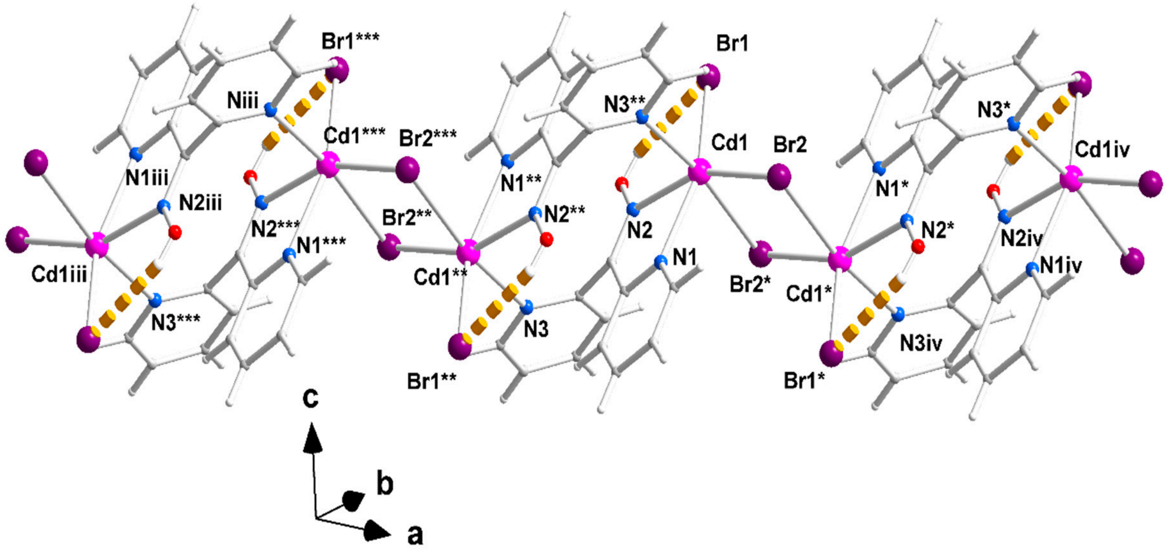

Figure S2. A portion of one zigzag chain (developed along the [1, 1, 0] crystallographic direction) of complex **2**. The thick dashed orange lines represent intrachain O_{oxime}-H···$Br_{terminal}$ H bonds. The symmetry codes (*), (**) and (***) are the same with those described in Figure 2b, and the additional codes are: (iii) 0.5-*x*, 0.5-*y*, 1-*z* and (iv) 0.5+*x*, 0.5+*y*, *z*.

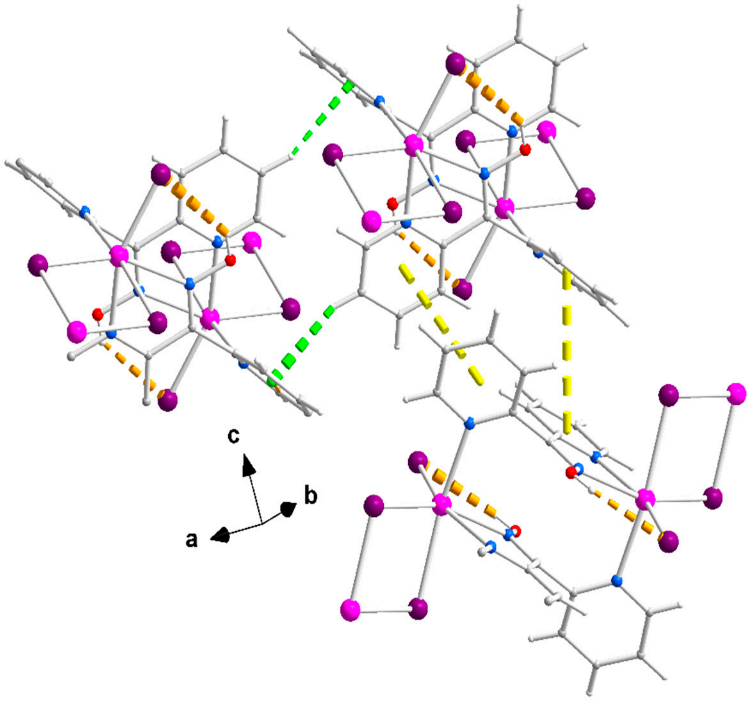

Figure S3. Interactions between neighboring chains in the crystal structure of compound **2**. The thick dashed light green and yellow lines indicate C2-H2···Cg1 (C-H···π) and π-π interactions, respectively; for details, see text. The thick dashed orange lines represent intrachain O_{oxime}-H···$Br_{terminal}$ H bonds (also see Figures 2b and 4).

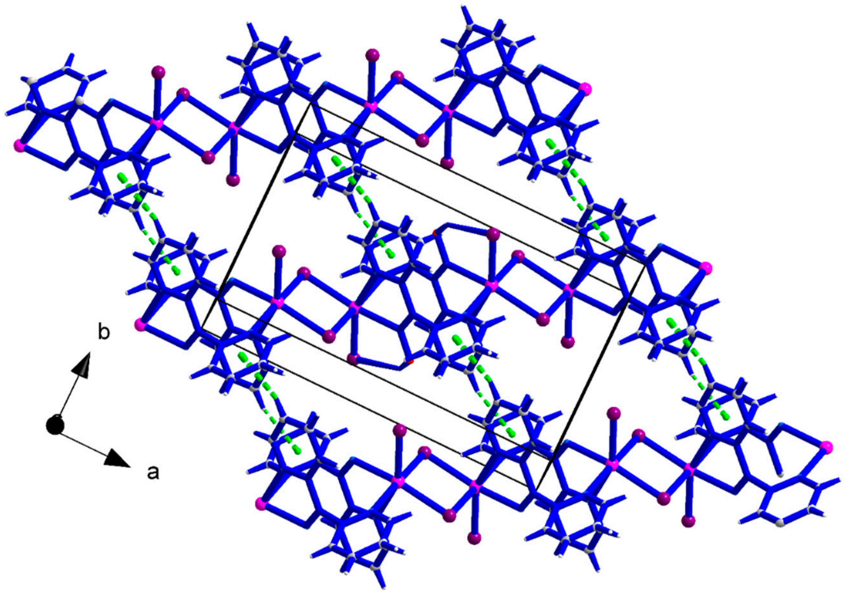

Figure S4. Layers of chains parallel to the (001) crystallographic planes in the structures of **2** and **3**. The chains are represented by thick blue solid lines and the C2-H2···Cg1 (C-H···π) interactions are indicated with thick dashed light green lines; for details, see text.

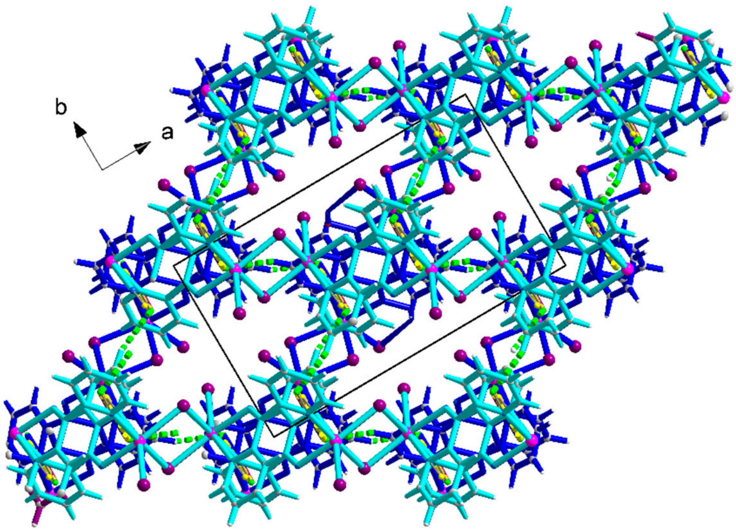

(a)

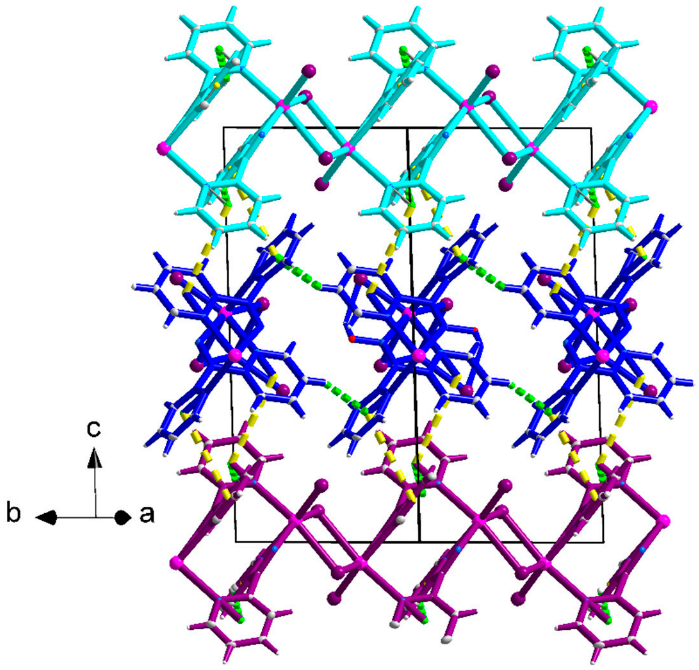

(b)

Figure S5. (a) Neighboring layers of **2** and **3** stacked along the *c* axis with a crossed-arrangement fashion relative to each other; in the top layer (cyan solid lines), the chains extend along the [-1, 1, 0] direction and in the bottom one (blue solid lines) along [1, 1, 0]. (b) Alternate stacking in **2** and **3** along the *c* axis; at the bottom (violet solid lines) and top (cyan solid lines) layers, the chains are along [-1, 1, 0] and in the middle one (blue solid lines) are along [1, 1, 0]. The thick dashed yellow and light green lines indicate C-H···π and π-π interactions; see also caption of Figure S2.

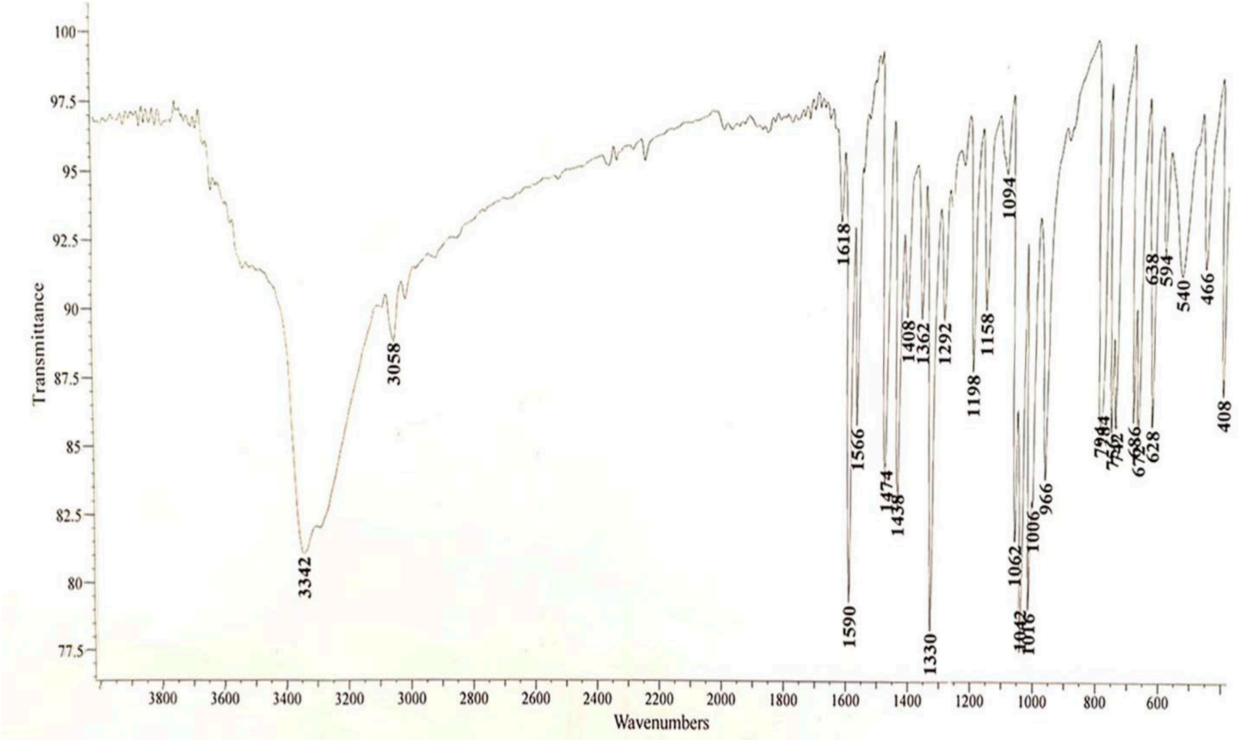

Figure S6. The IR spectrum (KBr, cm^{-1}) of the 1D polymer **2**.

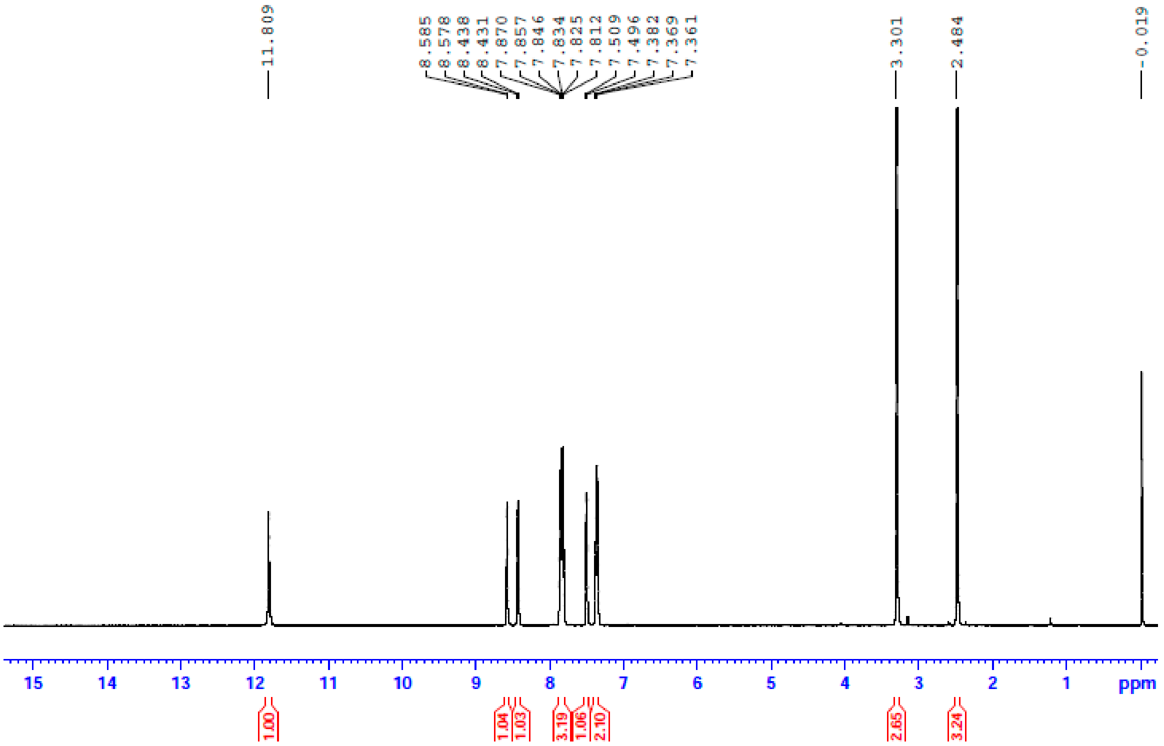

Figure S7. The ^{1}H NMR spectrum of **1**·$2H_2O$ in d_6-DMSO. The signal at δ 2.48 ppm is due to the methyl groups of the non-deuteriated amount of the solvent, and the signal at δ 3.30 ppm to the protons of the lattice H_2O and the water content of the solvent.

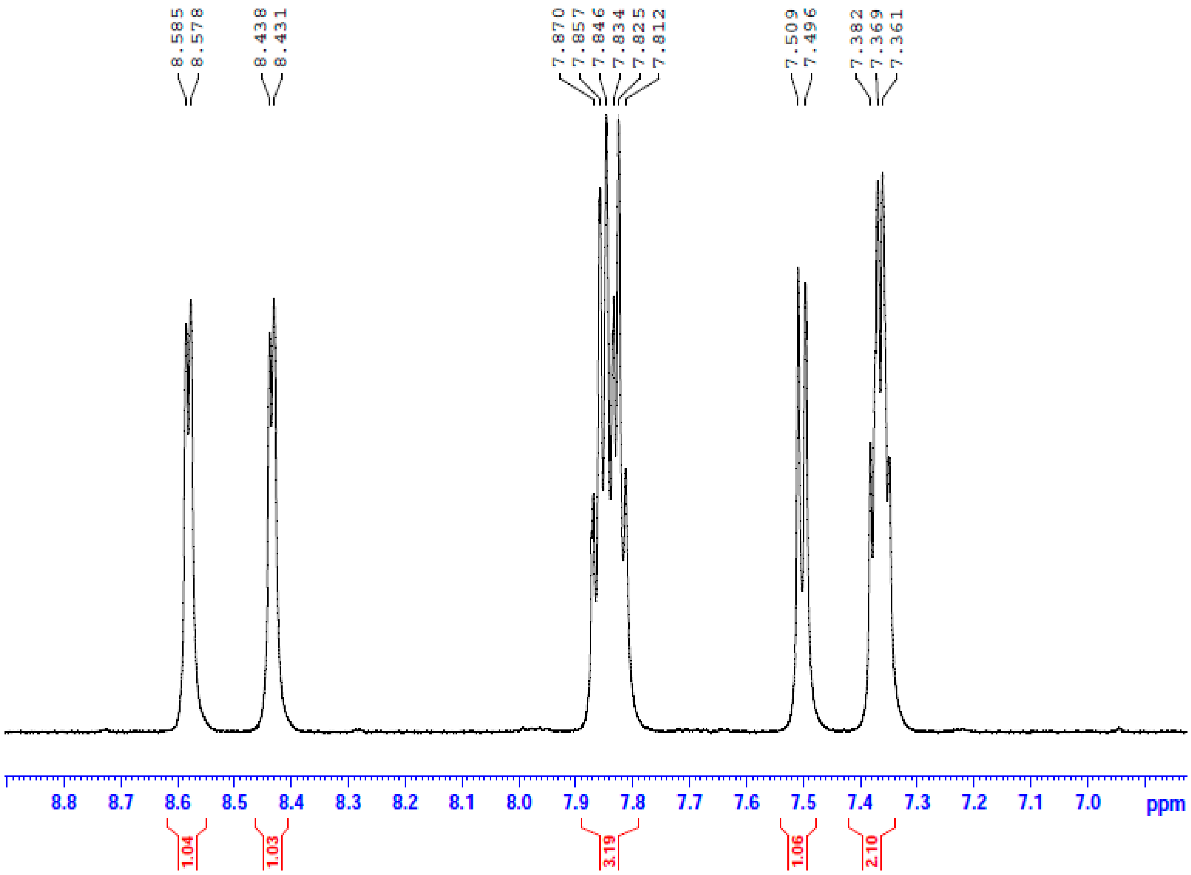

Figure S8. The ^{1}H NMR spectrum of **2** in d_6-DMSO in the aromatic region.
